# Quit Methods Used by American Smokers, 2013–2014

**DOI:** 10.3390/ijerph14111403

**Published:** 2017-11-17

**Authors:** Brad Rodu, Nantaporn Plurphanswat

**Affiliations:** 1 James Graham Brown Cancer Center, University of Louisville, 505 South Hancock Street, Louisville, KY 40202, USA; nantaporn.plurphanswat@louisville.edu; 2 Department of Medicine, School of Medicine, University of Louisville, Louisville, KY 40202, USA

**Keywords:** smoking cessation, e-cigarettes, pharmaceutical nicotine, NRT, quit-smoking attempts

## Abstract

This report describes the quit methods used in the past 12 months by current and former smokers in the baseline Population Assessment of Tobacco and Health (PATH) Study during 2013–2014. Descriptive statistics were used to report the use of single and two or more quit methods; survey weights were used to compute population estimates. Logistic regression was used to estimate the association between past year former smokers and single quit method, including individual characteristics. Results: Of 11,402 current smokers and 4919 former smokers, 4541 had tried and 839 had quit in the past 12 months. Unaided quit attempts were the most common; the number was almost as high as all single methods combined (n = 1797 and n = 1831 respectively). The most frequently used single method was help from friends and family (n = 676) followed by e-cigarettes (n = 587). Use of e-cigarettes was the only method with higher odds of users being a former smoker than unaided attempts (OR = 1.42, 95% CI 1.12–1.81). Current use of e-cigarettes among current (34%) and former (54%) smokers was significantly higher than current use of nicotine replacement therapy (NRT). Conclusions: In 2013–2014 e-cigarettes were used by American adult smokers as quit-smoking aids more frequently than NRT products or prescription drugs.

## 1. Introduction

The consumption of e-cigarettes expanded rapidly after 2010 in many countries, most notably in the United Kingdom and the United States. In the UK nearly 3 million people regularly use the products, and since 2013 they have been the most commonly used aid for smoking cessation [1]. Increasing prevalence of e-cigarette use in England has been positively associated with higher rates of successful quit attempts [2]. 

In the US, there is growing evidence from several nationally representative surveys that e-cigarette use is a frequently-used quit-smoking aid and enhances cessation rates. Analysis of the 2014–2015 Current Population Survey-Tobacco Use Supplement (CPS-TUS) revealed that smokers who used e-cigarettes were more likely than non-users to try to quit smoking and to succeed [3,4], and both outcomes were significantly associated with increasing frequency of e-cigarette use [4]. Another study using National Health Interview Survey (NHIS) data from the same years found that the prevalence of former smoking was significantly higher among daily e-cigarette users than among those who had never used them (52% vs. 28%), although some-day users were less likely to have quit (12%) [5]. In 2012–2013 only 1.9% of former smokers in the National Adult Tobacco Survey reported switching completely to e-cigarettes; in that survey one year later 3.8% of former smokers had switched [6]. 

The Centers for Disease Control and Prevention (CDC) reported that 20% of current smokers in the 2014 NHIS who had made a quit attempt had tried e-cigarettes [7]. Other investigators from the CDC used data from the Knowledge Panel Survey to report that current smokers more frequently used e-cigarettes than FDA-approved medications for quit attempts from 2014 to 2016 [8]. However, no specific information is available in these surveys about e-cigarette use before, during or after quit attempts by current and former smokers. In this report we extend the CDC’s observations by analyzing data from the Population Assessment of Tobacco and Health (PATH) Study.

The PATH Study is a combined project of the US Food and Drug Administration and the National Institutes of Health that enrolled two cohorts of adult and youth respondents in 2014. The objective of the PATH survey is to collect information on tobacco use patterns, risk perceptions and attitudes, and history of tobacco dependence and cessation for various tobacco products [9]. An overview of tobacco use among participants in the baseline PATH Wave 1 was recently published [10]. The purpose of this report is to describe the quit methods used in the past 12 months by current and former smokers in the baseline PATH survey. 

## 2. Materials and Methods

We used the Wave 1 Adult Public Use File from the PATH survey [11], which involved interviews with 32,320 civilian and non-institutionalized adults (ages 18 and older) from all 50 United States and the District of Columbia. Subject screening and enrollment from September 2013 to December 2014 used computer-assisted personal interviewing; interviews were completed with audio computer-assisted self-interviewing. PATH oversampled young adults (ages 18–24), blacks, and tobacco users. Information about the conceptual framework, methods [12] and sampling procedures [11] of the PATH survey are available. 

We used standard definitions for e-cigarette users and smokers. Respondents who had ever used e-cigarettes regularly and currently used them every day or some days were classified as current users. Current smokers had consumed at least 100 cigarettes in their lifetime and were smoking every day or some days at the time of the survey. Former smokers had smoked at least 100 cigarettes in their lifetime but were not smoking at the time of the survey. Both current and former smokers may currently use other tobacco products. Our analysis was restricted to current smokers who had tried to quit completely at least one time in the past 12 months and to past-year former smokers, because these were the only subgroups who were questioned about quit methods.

Past year quit methods were assessed by the following questions: “Thinking back to [the time you tried to quit (current smokers who tried one time) | the last time you tried to quit (current smokers who tried two or more times) | when you quit (former smokers)] cigarettes in the past 12 months,”

a “did you rely on the support of friends and family to help you?”
b “did you use any of the following to help you: counseling, a telephone help line or quit line, books, pamphlets, videos, a quit tobacco clinic, class, or support group, or an internet or web-based program?”
c “did you use any different tobacco product (e-cigarettes, traditional cigars, cigarillos, filtered cigars, pipe tobacco, hookah, snus pouches, smokeless tobacco, like dip, chew, or snuff, and dissolvable tobacco) to help you quit? Choose all that apply”
d “did you use a nicotine patch, gum, inhaler, nasal spray, lozenge or pill?” (hereafter nicotine replacement therapy, or NRT)
e “did you use Chantix, varenicline, Wellbutrin, Zyban, or bupropion?” (hereafter a prescription drug)

Data were analyzed by Stata, version 13 (College Station, TX, USA) using descriptive statistics with results for respondents reporting a single quit method and two or more methods. Population estimates were generated using PATH survey weights to reflect the U.S. adult population. In addition, we employed logistic regression to estimate the association, expressed as odds ratios (ORs), between past-year former smokers and single quit methods and other characteristics. We also estimated the odds of each method separately, coded as 1 if a respondent used it and 0 otherwise. These included: (1) did not use any quit method; (2) relied on support of friends and family; (3) used other methods (i.e., counseling, telephone help line or quit line, books, pamphlets, videos, quit tobacco clinic, class, or support group, internet or web-based program); (4) used e-cigarettes; (5) used other tobacco products (i.e., traditional cigars, cigarillos, filtered cigars, pipe tobacco, hookah, snus pouches, smokeless tobacco like dip, chew, or snuff); (6) used nicotine replacement products (i.e., patch, gum, inhaler, nasal spray, lozenge or pill); and (7) used a prescription drug (i.e., Chantix, varenicline; Wellbutrin, Zyban or bupropion). 

Individual characteristics (i.e., number of quit attempts, age, sex, race/ethnicity, education, household income, and region) were included as covariates in the regression analyses. We excluded participants with missing quit methods and individual characteristics. 

## 3. Results

The PATH dataset contains 11,402 current smokers and 4919 former smokers. Table 1 shows the derivation of the final numbers of current smokers who tried to quit smoking (n = 4541) and of former smokers who quit smoking (n = 839) in the past 12 months. Seventy-one percent of current smokers had tried to quit more than once, compared with 43% of former smokers. Thirty-four percent of current smokers had not used any method, 34% had used one method, 21% had used 2 methods and 8% had used 4 methods. The distribution according to number of methods used by former smokers was very similar.

Table 2 reveals the results for current and former smokers who did not use any quit method and those who used only one quit method. Unaided quitting attempts were the most common; the number was almost as high as the number of attempts with all single methods combined (n = 1797 and n = 1831 respectively). The most frequently used single method was help from friends and family (n = 676, 37% of all single methods) followed by e-cigarettes (n = 587, 32%), NRT (n = 338, 18%), prescription drugs (n = 106, 6%), counseling etc. (n = 49, 3%), other combustible products (n = 38, 2%) and snus/smokeless tobacco (n = 37, 2%). The nicotine patch was the most common NRT product used, and varenicline was used more frequently than bupropion.

Thirty-four percent of current smokers who had tried to quit with e-cigarettes were currently using them every day or some days at the time of the survey, which was significantly higher than the proportions for NRT (10%, z = 7.317, *p* < 0.0002) and a prescription drug (22%, z = 2.112, *p* = 0.0347). Among former smokers who had quit with e-cigarettes, 54% were currently using them, which was significantly higher than current use of NRT (29%, z = 2.853, *p* = 0.0043) but not of a prescription drug (35%, z = 1.71, *p* = 0.0873). Nicotine was consumed by the majority of current e-cigarette users. 

Table 3 has adjusted ORs for being a past-year former smoker, according to quit methods and other characteristics. Compared with smokers who did not use any quit method, the only method with significantly higher odds was use of e-cigarettes (OR = 1.43, 95% CI 1.12–1.83). Compared to 18–24 year olds, all older groups were significantly less likely to be a former smoker. Non-Hispanic Blacks were significantly less likely than Whites to be a former smoker, and higher education and income levels had the opposite effect.

Table 4 shows adjusted ORs for using each quit method, according to smoking status and other characteristics. The odds of using NRT and a prescription drug increased among older smokers. Men were more likely than women to use other tobacco products but less likely to rely on friends and family or use a prescription drug. Blacks and Hispanics were less likely than whites to use e-cigarettes. Education did not have prominent effects, but all higher income groups were more likely to use e-cigarettes. 

Table 5 shows the number of current and former smokers who used two or more quit methods. The rank order of frequency changed, with NRT replacing e-cigarettes as the second most common method (n = 969 and n = 957 respectively).

E-cigarettes were used as a single quit method by 2.2 million smokers in 2013–2014, NRT by 1.47 million, prescription drugs by 418,000 and smokeless tobacco by 124,000. Among smokers using multiple methods, NRT was used by 3.84 million smokers, e-cigarettes by 3.50 million, prescription drugs by 1.92 million and smokeless tobacco by 405,000. However, the latter estimates are not mutually exclusive. 

## 4. Discussion

Our analysis of the PATH survey shows that help from friends and family was the most common single quit method used in the past 12 months by American smokers in 2013–2014. Using e-cigarettes was the second most frequently used method, followed by NRT and a prescription drug. In addition, use of e-cigarettes was the only quit method producing significantly higher odds of being a past-year former smoker than using no method. E-cigarettes were also popular among smokers using two or more methods, although the results are not mutually exclusive in that group.

Comparison of population estimates for e-cigarettes, smokeless tobacco, NRT and prescription drugs are informative, because the first two are smoke-free tobacco products that are considered unacceptable as cigarette substitutes by government agencies and most American health authorities [13], while the latter two are universally promoted as safe and effective by the same.

In this study e-cigarettes were frequently used as a single quit method and were the only method with higher odds of former smoking than unassisted quitting. These results are consistent with studies from other national surveys including CPS-TUS [3,4], NHIS [5] and NATS [6]. Furthermore, these cohort studies suggest that e-cigarettes enhance smoking cessation at the population level in the absence of medical intervention, so the growing evidence complements positive results from clinical trials [14,15]. This study also meets some of the criteria recently proposed for studies related to e-cigarettes and smoking cessation, including a specific outcome (cessation), specific quit methods, and some information on extended use of those methods [16]. 

Among former smokers who had used e-cigarettes as a single quit method, 54% were still using them at the time of the survey. This is similar to the proportion of former smokers who had used smokeless tobacco to quit (46%), based on information in the 2000 NHIS [17], although the context and time frame in the latter survey were different. Almost half of former smokers who had quit with e-cigarettes had also discontinued vaping (46%, n = 59/128). Current use of NRT (29%) and prescription medications (35%) were also seen among former smokers, although the numbers of observations were small. These results may also be related to the time frame of the study, that is, in the past 12 months with no restriction for recency. Among current e-cigarette users, regardless of current or former smoking status, more than 90% were using products containing nicotine.

E-cigarette use has changed substantially in the 3–4 years since the PATH baseline survey was conducted. There were 8.9 million current vapers in 2014 the first year NHIS collected data on e-cigarettes [18]. By 2016 there were 7.8 million, one-third of whom (2.62 million) were former smokers. Although there were fewer current vapers in 2016 than in 2014, a higher proportion of them were former smokers in the latter year, which suggests that e-cigarette use remained a popular quit method.

This study confirms previous observations that quitting without any assistance is attempted by the largest number of smokers. Some tobacco control advocates believe that “an overly medicalised view of smoking cessation…sees unassisted cessation as both inefficient and inhumane” [19]. 

We view e-cigarettes as both a counter to the over-medicalisation of smoking cessation and as a necessary supplement for the persistent inadequacy of unassisted cessation. For these reasons we believe that smokers deserve full access to e-cigarettes and any other aids to become smoke-free, with or without nicotine/tobacco abstinence. 

There are some limitations with this study. This was an observational study using cross-sectional data. Smoking and quitting behaviors were self-reported and subject to recall error. There may be some instability in permanent cessation among former smokers who quit in the past 12 months. However, the PATH survey is designed to provide follow-up in subsequent waves over a longer period, which will inform tobacco researchers about patterns of cessation and regression. This study was not restricted to exclusive current and former smokers, but one of the quit methods asked by the PATH survey included switching to other tobacco products. Finally, there are some inconsistencies in reporting e-cigarette use; a small number of current smokers (4/459) reported that they have not seen or heard of e-cigarettes, but said that they used e-cigarettes to help them quit.

## 5. Conclusions

E-cigarettes were used by American adult smokers as quit-smoking aids in 2013–2014 more frequently than NRT products or prescription drugs. In addition, they were the only single quit method with a significantly higher proportion of former smokers than unaided quitting. 

## Figures and Tables

**Table 1:** Quit methods used in the past 12 months by current and former smokers, Population Assessment of Tobacco and Health (PATH) survey 2014.

	Current Smokers	Former Smokers
All	11,402	4919
Have tried to quit completely or have tried to quit by reducing or cutting back in the past 12 months	5572	n/a
Have tried to quit completely at least 1 time in the past 12 months	4593	n/a
Have completely quit smoking cigarettes in the past 12 months	n/a	1092
Number of quit methods used		
Did not use any method	1522	275
1 method	1529	302
2 methods	945	181
3 methods	374	61
4 methods	135	19
5 methods	32	1
6 methods	3	n/a
9 methods	1	n/a
Missing quit method	52	253
Final sample	4541	839

**Table 2:** Numbers and percentages of current and former smokers using a single or no quit method in the past 12 months.

Quit Methods	Current Smokers				Former Smokers	
	Every Day		Some Day			
	N	(%)	N	(%)	n	(%)
1. Used no quit method	**1006**	**47.1**	**516**	**44.9**	**275**	**47.7**
2. Relied on support of friends and family	**399**	**18.7**	**182**	**15.8**	**95**	**16.5**
3. Used other methods ^1^	**30**	**1.4**	**13**	**1.1**	**6**	**1.0**
4. Used other tobacco products	**390**	**18.3**	**129**	**11.2**	**143**	**24.8**
4.1. E-cigarettes	351		108		128	
Not aware of e-cigarettes	3		1		n/a	
Do not currently use e-cigarettes	248		53		59	
Currently use e-cigarettes	100		54		69	
With nicotine	97		51		62	
Without nicotine	3		3		7	
4.2. Combustible tobacco products ^2^	21		10		7	
4.3. Smokeless tobacco ^3^	18		11		8	
4.4. Dissolvable tobacco	0		0		0	
5. Used NRT products	**235**	**11.0**	**61**	**6.7**	**42**	**7.3**
5.1. Nicotine patch	159		32		21	
5.2. Nicotine gum	83		18		11	
5.3. Others ^4^	19		14		11	
5.4. None of above	9		6		3	
Do not currently NRT	223		43		29	
Currently use NRT	12		18		12	
6. Used a prescription drug	**74**	**3.5**	**16**	**1.7**	**16**	**2.8**
6.1. Chantix or varenicline	45		7		8	
6.2. Wellbutrin, Zyban or bupropion	31		10		6	
6.2. None of above	2		1		2	
Do not currently use a prescription drug	59		11		11	
Currently use a prescription drug	15		5		5	
**Quit methods in total**	**2134**	**100.0**	**917**	**100.0**	**577**	**100.0**

Bold text and numbers are quit methods that contribute to column totals. ^1^ counseling, a telephone help line or quit line, books, pamphlets, videos, a quit tobacco clinic, class, or support group, or an internet or web-based program. ^2^ traditional cigars, cigarillos, filtered cigars, pipe tobacco, hookah. ^3^ snus pouches, smokeless tobacco like dip, chew, or snuff. ^4^ inhaler, nasal spray, lozenge or pill.

**Table 3:** Adjusted odds ratios (OR) and 95% confidence intervals (CI) for being a past-year former smoker, according to single quit method and other characteristics.

Quit Methods	OR [95% CI]
Did not use any quit method	Reference
Relied on support of friends and family	0.98 [0.75,1.28]
Used other methods ^1^	0.89 [0.36,2.17]
Used e-cigarettes	**1.43 [1.12,1.83]**
Used other tobacco products ^2^	1.43 [0.78,2.63]
Used NRT products ^3^	0.89 [0.61,1.28]
Used a prescription drug ^4^	0.97 [0.55,1.71]
Number of quit attempts	
1	Reference
2–3	**0.38 [0.31,0.46]**
4+	**0.28 [0.21,0.37]**
Age groups (years)	
18–24	Reference
25–44	**0.78 [0.62,0.98]**
45–64	**0.71 [0.54,0.94]**
65 and older	0.64 [0.38,1.06]
Sex	
Female	Reference
Male	0.88 [0.72,1.06]
Race/ethnicity	
Non-Hispanic White	Reference
Non-Hispanic Black	**0.70 [0.50,0.97]**
Hispanic	0.92 [0.70,1.22]
Non-Hispanic other race	0.83 [0.58,1.17]
Education	
Less than high school	Reference
High school or GED	1.31 [0.95,1.84]
Some college	**1.65 [1.19,2.30]**
College and more	**1.92 [1.29,2.87]**
Household income	
Less than $10,000	Reference
$10,000 to $24,999	0.82 [0.61,1.10]
$25,000 to $49,999	0.99 [0.73,1.34]
$50,000 to $99,999	1.16 [0.83,1.61]
More than $100,000	1.45 [0.97,2.16]
Missing income	1.41 [0.96,2.08]
Region	
Northeast	Reference
South	0.98 [0.72,1.35]
West	1.40 [1.02,1.94]
Midwest	1.18 [0.88,1.59]
Observations 5	3561

Bold indicates statistically significant estimates (*p* < 0.05). ^1^ counseling, telephone help line or quit line, books, pamphlets, videos, quit tobacco clinic, class, or support group, internet or web-based program. ^2^ traditional cigars, cigarillos, filtered cigars, pipe tobacco, hookah. ^3^ snus pouches, smokeless tobacco like dip, chew, or snuff. ^4^ inhaler, nasal spray, lozenge or pill. ^5^ Excluding those who had missing quit methods and other characteristics.

**Table 4:** Adjusted odds ratios (OR) and 95% confidence intervals (CI) for using each single quit method, according to smoking status and other characteristics.

	Did not Use Any Quit Method	Relied on Support of Friends and Family	Used Other Methods ^1^	Used E-Cigarettes	Used Other Tobacco Products ^2^	Used NRT Products ^3^	Used a Prescription Drug ^4^
	OR [95% CI]						
Smoking status							
Current smokers	Reference	Reference	Reference	Reference	Reference	Reference	Reference
Past year former smokers	0.88 [0.73,1.06]	0.91 [0.71,1.17]	0.85 [0.35,2.05]	**1.43 [1.14,1.81]**	1.43 [0.79,2.61]	0.83 [0.58,1.19]	0.90 [0.51,1.59]
Number of quit attempts							
1	Reference	Reference	Reference	Reference	Reference	Reference	Reference
2–3	**0.76 [0.65,0.89]**	**1.45 [1.18,1.78]**	1.52 [0.76,3.03]	0.90 [0.73,1.10]	1.70 [1.00,2.89]	1.18 [0.89,1.57]	0.98 [0.62,1.54]
4 or more	0.83 [0.69,1.01]	1.34 [1.05,1.71]	1.20 [0.52,2.77]	0.91 [0.71,1.18]	0.70 [0.30,1.46]	1.30 [0.94,1.79]	0.86 [0.49,1.51]
Age groups (years)							
18–24	Reference	Reference	Reference	Reference	Reference	Reference	Reference
25–44	**0.82 [0.69,0.97]**	0.90 [0.73,1.11]	0.70 [0.34,1.42]	1.25 [1.00,1.57]	0.77 [0.47,1.26]	**1.95 [1.34,2.83]**	**2.81 [1.40,5.68]**
45–64	**0.80 [0.66,0.96]**	**0.69 [0.54,0.89]**	0.96 [0.45,2.05]	1.00 [0.77,1.31]	**0.15 [0.05,0.42]**	**4.15 [2.86,6.03]**	**4.27 [2.08,8.75]**
65 and older	1.06 [0.77,1.46]	**0.32 [0.18,0.55]**	0.95 [0.26,3.42]	**0.50 [0.28,0.87]**	n/a	**5.73 [3.54,9.28]**	**7.19 [3.02,17.08]**
Sex							
Female	Reference	Reference	Reference	Reference	Reference	Reference	Reference
Male	**1.15 [1.01,1.32]**	**0.69 [0.58,0.83]**	0.84 [0.48,1.52]	1.01 [0.84,1.22]	**3.98 [2.26,7.00]**	1.18 [0.93,1.49]	**0.55 [0.36,0.83]**
Race/ethnicity							
Non-Hispanic White	Reference	Reference	Reference	Reference	Reference	Reference	Reference
Non-Hispanic Black	**1.45 [1.18,1.77]**	0.93 [0.71,1.21]	1.68 [0.80,3.53]	**0.51 [0.37,0.71]**	1.26 [0.65,2.42]	0.98 [0.70,1.36]	0.50 [0.25,1.00]
Hispanic	**1.46 [1.20,1.78]**	1.12 [0.88,1.43]	1.03 [0.43,2.46]	**0.68 [0.51,0.90]**	0.62 [0.29,1.31]	**0.59 [0.40,0.89]**	**0.37 [0.16,0.86]**
Non-Hispanic other race	1.11 [0.87,1.42]	0.91 [0.66,1.26]	0.86 [0.26,2.88]	1.07 [0.78,1.48]	1.13 [0.49,2.56]	0.85 [0.54,1.35]	0.74 [0.34,1.64]
Education							
Less than high school	Reference	Reference	Reference	Reference	Reference	Reference	Reference
High school or GED	1.18 [0.96,1.45]	0.85 [0.66,1.09]	0.90 [0.38,2.14]	1.15 [0.85,1.57]	0.64 [0.31,1.31]	**0.69 [0.49,0.97]**	1.92 [0.91,4.05]
Some college	1.17 [0.95,1.45]	**0.69 [0.53,0.90]**	1.01 [0.42,2.40]	1.26 [0.92,1.71]	1.10 [0.56,2.16]	0.76 [0.54,1.07]	1.80 [0.84,3.86]
College and more	**1.36 [1.03,1.79]**	0.74 [0.51,1.06]	0.74 [0.21,2.66]	1.00 [0.68,1.49]	0.58 [0.21,1.66]	0.81 [0.51,1.27]	1.51 [0.60,3.78]
Household income							
Less than $10,000	Reference	Reference	Reference	Reference	Reference	Reference	Reference
$10,000 to $24,999	0.84 [0.69,1.02]	0.97 [0.76,1.23]	1.19 [0.52,2.73]	**1.85 [1.38,2.50]**	0.96 [0.49,1.84]	0.81 [0.56,1.15]	0.78 [0.42,1.47]
$25,000 to $49,999	0.89 [0.72,1.09]	0.92 [0.71,1.20]	1.08 [0.43,2.71]	**1.79 [1.31,2.44]**	0.54 [0.24,1.19]	0.85 [0.58,1.23]	0.92 [0.49,1.72]
$50,000 to $99,999	0.79 [0.62,1.00]	0.91 [0.67,1.23]	1.88 [0.74,4.80]	**1.71 [1.21,2.40]**	1.20 [0.57,2.52]	0.98 [0.65,1.48]	0.96 [0.48,1.91]
More than $100,000	0.86 [0.63,1.18]	0.65 [0.42,1.01]	0.42 [0.05,3.46]	**2.04 [1.34,3.09]**	0.80 [0.27,2.34]	1.10 [0.65,1.86]	1.05 [0.44,2.51]
Missing income	1.10 [0.82,1.46]	0.61 [0.41,0.91]	0.88 [0.24,3.26]	1.19 [0.76,1.86]	0.57 [0.19,1.74]	1.49 [0.95,2.32]	0.86 [0.36,2.05]
Region							
Northeast	Reference	Reference	Reference	Reference	Reference	Reference	Reference
South	0.95 [0.77,1.18]	1.08 [0.82,1.43]	0.97 [0.42,2.24]	1.10 [0.81,1.49]	0.68 [0.32,1.43]	0.90 [0.63,1.27]	1.30 [0.69,2.48]
West	1.08 [0.85,1.36]	1.02 [0.76,1.38]	0.62 [0.23,1.71]	1.12 [0.81,1.55]	0.57 [0.25,1.32]	0.79 [0.53,1.18]	1.02 [0.49,2.12]
Midwest	1.04 [0.85,1.27]	0.92 [0.70,1.19]	0.68 [0.30,1.56]	1.31 [0.98,1.75]	0.91 [0.47,1.77]	0.71 [0.51,1.00]	1.17 [0.62,2.19]
Observations ^5^	3561	3561	3561	3561	3561	3561	3561

Bold indicates statistically significant estimates (*p* < 0.05). n/a—not available; the number of survey respondents in this group was 0. ^1^ counseling, telephone help line or quit line, books, pamphlets, videos, quit tobacco clinic, class, or support group, internet or web-based program. ^2^ traditional cigars, cigarillos, filtered cigars, pipe tobacco, hookah, snus pouches, smokeless tobacco like dip, chew, or snuff. ^3^ patch, gum, inhaler, nasal spray, lozenge or pill. ^4^ Chantix, varenicline; Wellbutrin, Zyban or bupropion. ^5^ excluding those who had missing quitting methods and other characteristics.

**Table 5:** Numbers of current and former smokers using two or more quit methods in the past 12 months.

Quit Methods	Current Smokers		Former Smokers
	Every Day	Some Day	
1. Relied on support of friends and family	845	186	186
2. Used other methods ^1^	326	77	38
3. Used other tobacco products	812	200	200
3.1. E-cigarettes	641	148	168
Not aware of e-cigarettes	3	0	n/a
Do not currently use e-cigarettes	453	76	78
Currently use e-cigarettes	187	72	90
With nicotine	174	68	86
Without nicotine	12	4	4
3.2. Combustible tobacco products ^2^	88	27	13
3.3. Smokeless tobacco ^3^	74	20	18
3.4. Dissolvable tobacco	9	5	1
4. Used NRT products	718	121	130
4.1. Nicotine patch	449	74	63
4.2. Nicotine gum	210	32	34
4.3. Others ^4^	154	27	29
4.4. None of above	66	11	19
Do not currently NRT	665	85	99
Currently use NRT	52	36	30
5. Used a prescription drug	351	59	66
5.1. Chantix or varenicline	244	38	46
5.2. Wellbutrin, Zyban or bupropion	124	25	24
5.3. None of above	8	1	0
Do not currently use a prescription drug	292	37	50
Currently use a prescription drug	59	22	16

^1^ counseling, a telephone help line or quit line, books, pamphlets, videos, a quit tobacco clinic, class, or support group, or an internet or web-based program. ^2^ traditional cigars, cigarillos, filtered cigars, pipe tobacco, hookah. ^3^ snus pouches, smokeless tobacco like dip, chew, or snuff. ^4^ inhaler, nasal spray, lozenge or pill.
